# Use of Autologous Mesenchymal Stem Cells Derived from Bone Marrow for the Treatment of Naturally Injured Spinal Cord in Dogs

**DOI:** 10.1155/2014/437521

**Published:** 2014-02-25

**Authors:** Euler Moraes Penha, Cássio Santana Meira, Elisalva Teixeira Guimarães, Marcus Vinícius Pinheiro Mendonça, Faye Alice Gravely, Cláudia Maria Bahia Pinheiro, Taiana Maria Bahia Pinheiro, Stella Maria Barrouin-Melo, Ricardo Ribeiro-dos-Santos, Milena Botelho Pereira Soares

**Affiliations:** ^1^Centro de Pesquisas Gonçalo Moniz, Fundação Oswaldo Cruz, 40296-710 Salvador, BA, Brazil; ^2^Hospital de Medicina Veterinária, Escola de Medicina Veterinária e Zootecnia, Universidade Federal da Bahia, 40170-110 Salvador, BA, Brazil; ^3^Departamento de Ciências da Vida, Universidade do Estado da Bahia, 41150-000 Salvador, BA, Brazil; ^4^Hospital Espanhol, 40140-110 Salvador, BA, Brazil; ^5^A Arca Veterinária, 40243-045 Salvador, BA, Brazil; ^6^Estácio-FIB, Centro Universitário Estácio da Bahia, 41770-030 Salvador, BA, Brazil; ^7^Centro de Biotecnologia e Terapia Celular, Hospital São Rafael, 41253-190 Salvador, BA, Brazil

## Abstract

The use of stem cells in injury repair has been extensively investigated. Here, we examined the therapeutic effects of autologous bone marrow mesenchymal stem cells (MSC) transplantation in four dogs with natural traumatic spinal cord injuries. MSC were cultured *in vitro*, and proliferation rate and cell viability were evaluated. Cell suspensions were prepared and surgically administered into the spinal cord. The animals were clinically evaluated and examined by nuclear magnetic resonance. Ten days after the surgical procedure and MSC transplantation, we observed a progressive recovery of the panniculus reflex and diminished superficial and deep pain response, although there were still low proprioceptive reflexes in addition to a hyperreflex in the ataxic hind limb movement responses. Each dog demonstrated an improvement in these gains over time. Conscious reflex recovery occurred simultaneously with moderate improvement in intestine and urinary bladder functions in two of the four dogs. By the 18th month of clinical monitoring, we observed a remarkable clinical amelioration accompanied by improved movement, in three of the four dogs. However, no clinical gain was associated with alterations in magnetic resonance imaging. Our results indicate that MSC are potential candidates for the stem cell therapy following spinal cord injury.

## 1. Introduction

Traumatic spinal cord injury (SCI) has become a more frequent condition, with an annual worldwide incidence of 15 to 40 cases per million [1]. SCI has a major negative impact on functional, medical, and financial aspects of the injured person, as well as deleterious effect on the individual's psychosocial well-being [2–4]. The pathophysiology of SCI is determined not only by the initial mechanical insult but also by secondary processes, which include ischemia, anoxia, free-radical formation, and cytotoxicity that can occur in the immediate hours to days following injury. Lesion evolution following SCI involves neuronal death by both necrosis and apoptosis [5], and since regeneration of the central nervous system is limited after injuries, it is crucial to develop novel approaches that optimize functional recovery after SCI. Potential therapies may include strategies to reduce the progression of secondary injuries, modulate the microenvironment of the spinal cord, remyelination and improve the intrinsic regenerative potential of endogenous progenitor cells [6].

Recently, cell transplantation has been considered a promising option for the treatment of neuronal disorders [7], including spinal cord lesions [8–10]. The initial pioneering studies most frequently involved neural stem cells as the desired cell source explored for regeneration of nervous system [11]. However, due to their limited availability, alternative sources for neural cell transplantation, such as embryonic, bone marrow, adipose, and umbilical cord blood stem cells, have also been investigated [7, 9, 12–16]. The cells obtained from these additional sources have been shown to survive, proliferate, migrate, and differentiate into neuronal phenotypes in the damaged brain and spinal cord [8, 17–19]. Despite positive results obtained in experimental models using a variety of animal species and recent dramatic progress in cellular transplantation, development of powerful strategies to treat SCI remains a major clinical challenge [8, 20].

Naturally, injured dogs are considered excellent models for the comparative study and comprehension of human diseases, which includes nervous system pathologies, due to the fact that they present similar anatomical, physiological, and immunological characteristics. Dogs and humans share innumerous genetic, traumatic, infectious, and metabolic diseases. The most prevalent causes of spinal cord lesion in dogs are car accidents and degenerative disk diseases, occurring at or near the thoracolumbar junction and producing chronic, complete paraplegia [21]. The damaged disks can burst or bulge and exert pressure on the delicate spinal cord, interrupting the spinal blood supply and is considered a traumatic SCI that induces a wide range of pathological events, generally resulting in a permanent state of sensory and motor loss [22, 23]. In addition to these well-described pathological alterations, traumatic SCI can cause severe deficits within the urogenital system [24, 25] and can include conditions that are both chronic and potentially life-threatening as well as cause a significant reduction in human quality of life [26] and in companion animals [21, 27, 28].

Currently there is no consensual medical therapy standard for the treatment of spinal cord injury in dogs. Practiced medical therapies aim to control secondary injury mechanisms (e.g., free radical scavenging) that occur following the primary injury insult. No satisfactory curative therapy has been accepted for chronic cases in both dogs and humans.

In the present study, we have isolated and cultivated *in vitro* MSC obtained from bone marrow of dogs suffering from chronic SCI due to degenerative disc disease. These primary adult mesenchymal stem cell cultures were characterized and applied within the injured spinal cord. The animals were clinically monitored during 18 months for safety and evaluation of the cell therapy efficacy.

## 2. Materials and Methods

### 2.1. Animals and Ethics

Following a formal agreement with the animal owners, implementation of welfare observation, and biological security protocols, 12 dogs presenting spinal cord compression due to herniated intervertebral disks (T12 to L5 vertebrae) were submitted to decompressive surgery (hemilaminectomy). Eight animals presented motor gains during follow-up evaluations. Four adult dogs presenting chronic unfavorable follow-up evaluations (no neurological gain for 6 months after surgery) were included in this study (Table 1). Animals were subjected to physical therapy and clinical evaluation for 6 months prior to and during the 12 months following MSC administration. All dogs were scored according to the degree of clinical neurological dysfunction and by nuclear magnetic resonance at two time points: (1) day of cell transplantation and (2) at the conclusion of the follow-up evaluation (18 months). A radiographic exam was performed in order to confirm the location of spinal cord lesion and to exclude other diseases.

All animal procedures performed were in accordance with guidelines defined by the Committee of Ethics in Animal Experimentation of the HSR—CEUA, Bahia, Brazil, and in agreement with ethical publications [21, 28].

### 2.2. Bone Marrow Isolation and Cell Culture

Bone marrow aspiration in dogs was performed by puncturing the iliac crest under sedation with diazepam (0.5 mg/kg/iv) associated with tramadol hydrochloride (1.0 mg/kg/iv) in lateral decubitus. After shaving and asepsis at the site for puncture, 0.5 to 1.0 mL of bone marrow was collected in previously heparinized syringes. Collected samples were diluted in Dulbecco's modified Eagle's medium, DMEM (Gibco, Carlsbad, CA, USA), and the fraction with mononuclear cells was obtained by Ficoll-Hypaque gradient (Sigma, St Louis, MO, USA), after centrifugation at 400 ×g for 30 minutes at 20°C. The interface containing mononuclear cells was collected in individualized tubes and washed twice in incomplete DMEM. The cells were cultured, as previously described [29, 30]. Briefly, mononuclear cells were resuspended in DMEM medium supplemented with 2 mM L-glutamine, 1 mM sodium piruvate, 50 *μ*g/mL gentamycin, and 10% fetal bovine serum (all reagents were purchased from Sigma) and cultured at the density of 10^5^ cells/cm^2^ in polystyrene plates. Cell cultures were maintained at 37°C with 5% CO_2_. When 80% confluence was reached, cells were detached using 0.25% trypsin (Invitrogen/Molecular Probes, Eugene, OR, USA) and expanded in new culture bottles (9 × 10^3^ cells/cm^2^). The cells were expanded during approximately 5 to 10 passages and aliquots were frozen to be utilized in different stages. Cell viability was determined by trypan blue exclusion test.

### 2.3. Proliferation Assay

Carboxyfluorescein diacetate succinimidyl ester (CFSE) assay (Invitrogen/Molecular Probes) was performed according to methodology described previously [31], following the manufacturer instructions. Acquisition and analysis were conducted using a FACScalibur cytometer (Becton Dickinson, San Diego, CA, USA) with CellQuest software. A minimum of 50,000 events were collected.

### 2.4. Differentiation Assays

The potential to differentiate into osteogenic and adipogenic lineages was examined. To promote osteogenesis, the cells were incubated with DMEM, supplemented with 10 mM *β*-glycerol phosphate (Sigma), 0.05 mM ascorbate-2-phosphate (Sigma), and 100 nM dexamethasone (Sigma). The culture medium was changed two times per week for up to three weeks. To detect calcium, the cells were fixed with methanol for 10 minutes at room temperature and stained with alizarin red at pH 4.0 for 5 minutes at room temperature. For adipogenesis, cultured cells were incubated in adipogenic medium that included DMEM, supplemented with 60 mM indomethacin (Sigma), 0.5 mM hydrocortisone (Sigma), and 0.5 mM isobutylmethylxanthine (Sigma). The culture medium was changed two times per week for up to three weeks. The cells then were fixed in methanol for 45 minutes and stained with Oil Red (Sigma) for detection of lipid accumulation.

### 2.5. Clinical Evaluation

**Figure 1:**
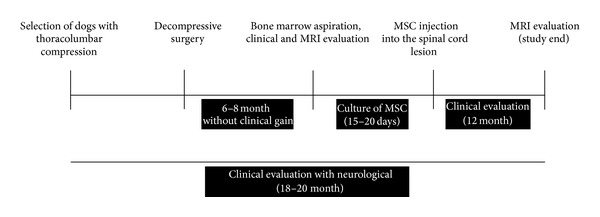
Data acquisition schematic and methods.

The dogs were clinically, radiographically, and by nuclear magnetic resonance imaging (MRI) evaluated before and after the cell transplantation (Figure 1, Tables 2 and 3). Prior to surgery, different degrees of superficial and deep pain deficit, muscular tone, and panniculus reflexes from the lesioned area to the nail edge of both caudal limbs were observed. Physical exams were performed according to [28]. The observed clinical signs were (a) patellar and sciatic reflexes; (b) dorsal panniculus reflex; (c) pain reflex; and (d) anterior and posterior weight bearing limbs. Conscious proprioception was the first test to be performed by inverting each paw, one at a time, so that the animal was standing upon the dorsum of the foot and was classified as “positive” when the animal almost immediately repositioned the foot to the normal position and “negative” when it did not respond within 15 seconds.

Superficial pain was tested by pinching the webbing between the toes, while deep pain was tested by clamping a 16 cm universal hemostat on the joints of the digits to periosteum stimulation, where limb withdrawal represented the expected spinal reflex. Stimulation of the lateral spinothalamic tract and subsequent transfer of information to the cerebral cortex should result in a behavioral response, such as crying, snapping or changing position, or autonomic activities. If one or more of these behavioral responses were observed, it was classified as deficiency of pain.

Reflex examinations were performed with the animal positioned on its side allowing for the muscle to be relaxed. The patellar tendon was then struck with a reflex hammer and the muscle reaction was observed. Both posterior limbs were checked, although the “free” or “up limb” was the only limb recorded. The reflexes were graded from 0 to 4 based upon the response: 0 = areflexia; 1 = diminished reflex; 2 = normal reflex; 3 = hyperactive reflex; and 4 = hyperactive reflex with clonus. The posterior limb reflexes tested were patellar (segments L4-L5); cranial tibialis (segments L6-S2), and sciatic notch (segments L6-S2).

Two other subjective criteria were also evaluated: tail movement and urinary bladder voluntary control. The neurological gain of both aspects was classified as “no,” “partial,” and “total” considering conscious movements of the tail and bladder continence. Neurologic function was characterized by use of a modified Frankel score (MFS; determined on a scale of 0 to 5, where 0 represented paraplegia with no deep nociception and 5 represented paraspinal hyperesthesia only) [32]. Long-term follow-up evaluations were later assessed.

*Imaging Assessment*. a nuclear magnetic resonance imaging (MRI equipment: magnetom aera 1.5T, Siemens, USA) evaluation was performed before MSC administration to evaluate the morphologic aspect of spinal cord. The acquisition was performed on anesthetized dogs that were positioned in dorsal recumbence with limbs flexed along the torso. T1, T2, and short tau inversion recovery sequences were acquired by spin echo as well as by using the more rapid single shot fast spin echo and gradient echo pulse sequences. Three large overlapping fields of view (FOV) were used to visualize the thoracic-lumbar spine, which was compared to the sagittal and dorsal plane images.

### 2.6. Surgical Procedure and MSC Administration

Each animal underwent a second hemilaminectomy in the same location as the initial decompression surgery (before 6 months), under general anesthesia (induction: propofol 8.0 mg/kg, maintenance: 2.0 to 2.5% isoflurane with oxygen 100%) followed by autologous MSC therapy. Cell preparations with more than 96% of viability were used in the transplant. Initially, a 1.0 mL syringe with a 13 × 4.5 mm needle was used to inject DMSO (0.2 mL/cm^3^ of lesion) into the injured area. The administration of 10^6^ cells in each 1.0 cm^3^ of lesion was performed continuously along 5 mm of the central dorsal spine. Bovine collagen gelfoam (Pfizer, Portage, MI, USA) was used as a scaffold over the administration area to prevent cell escape.

### 2.7. Pain Control

The pain was controlled by the use of tramadol 2 mg/kg/TID for 4 days (Dorless V—Agener União, Pouso Alegre, Brazil) and meloxicam 0.2 mg/Kg prior to surgery and 0.1 mg/kg/SID for 5 days (Maxican—Ouro fino, Cravinho, Brazil), before and after surgery. The dogs were maintained and monitored in the hospital for 2 days and, according to individual needs, received an extra pharmacological pain relief and physical therapy, which included spine mobilization/manipulation, therapeutic exercise, neuromuscular reeducation, hot/cold packs, and electrical muscle stimulation. Active kinesiotherapy involved dog treadmill training.

## 3. Results

### 3.1. Morphological Features, Proliferation, Viability, and Differentiation of the Mesenchymal Bone Marrow Stem Culture Cells

**Figure 2:**
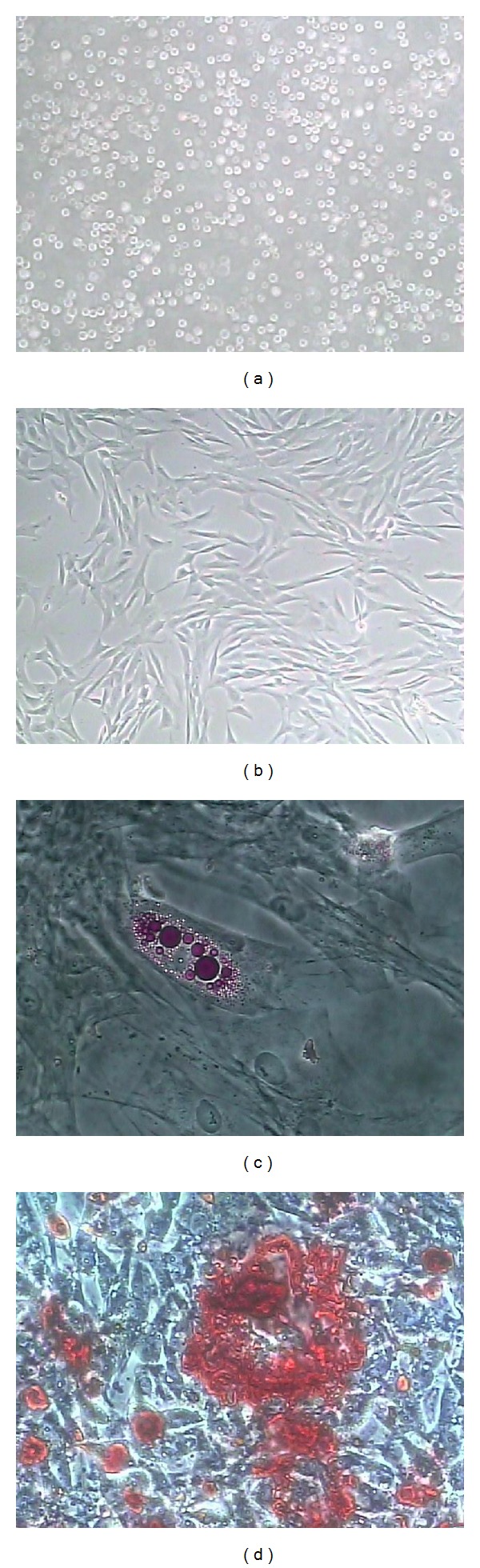
Collection, culture, differentiation, and application of dog bone marrow mesenchymal stem cells (MSC), in dog 2. (a) and (b) control cell cultures in noninducing medium. (a) Day one of a first passage culture. (b) Cell confluence after 15 days of culture. (c) Adipogenic differentiation; Oil red staining showing cytoplasmatic fat granuli inside cells. (d) Osteogenic differentiation. Alizarin red staining showing mineralized matrix deposition within stem cells. Magnification: (a) and (b) 100X; (c) and (d) 400X.

Canine mesenchymal stem cells obtained from bone marrow were adherent to the polystyrene culture flasks and showed rapid expansion and proliferation after *in vitro* isolation. Each bone marrow aspiration procedure yielded on average 3.5 × 10^7^ mononuclear cells. When maintained in culture, these cells presented fibroblast-like and rounded morphologies (Figure 2(a)). Cell confluence was obtained after 15 days of culture (Figure 2(b)). Quantitative evaluation of the exponential cell expansion phase was estimated by CFSE staining. Approximately, 80% of the canine MSC proliferated after 24 hours of culture (Figure 2(a)). In addition, MSC presented differentiation potential to form osteocytes and adipocytes after two weeks of culture using specific differentiation induction mediums (Figures 2(c) and 2(d)).

### 3.2. Clinical Findings

Neurological recovery after the decompression varied greatly, being inversely proportional to the duration of compression. We observed low to absent neurological recovery after 72 hours of compression and no degree of recovery after 96 hours of compression. Dogs displaying significant neurological recovery 6 months after surgery were not submitted to cell therapy.

**Figure 3:**
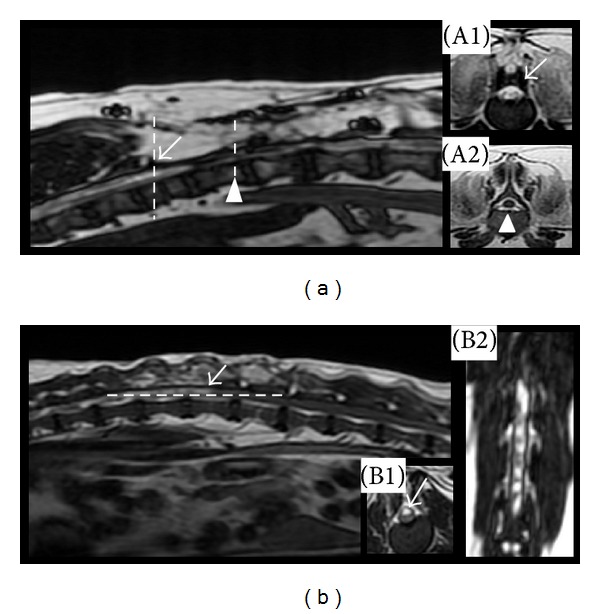
(a) MRI of a dog with syringomyelia (dog 4) showing area of hypersignal ((A1): white arrow) indicating spinal cord degeneration and an area void of spinal cord presence signal ((A2): arrow head). (b) MRI of a dog (dog 3) with a hypersignal (white arrow), indicating spinal cord degeneration also within axial (B1) and dorsal (B2) aspects regions.

**Figure 4:**
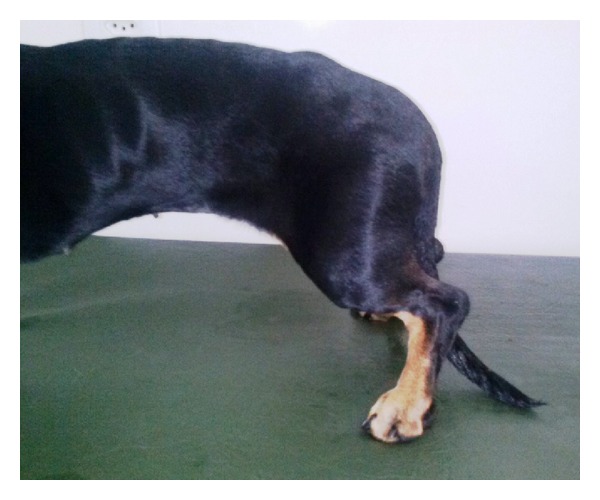
First minute weight bearing of dog (dog 2) 100 days after surgery.

Regarding the dogs that underwent cell therapy, from the first to the ninth day after MSC transplantation, we did not observe differences in the state of the medullar reflexes, including superficial and deep nociception reflexes, in comparison to the clinical condition of all four dogs before the intervention. On the 10th day, however, a progressive recovery of panniculus reflex and superficial and deep pain were observed, despite the fact that proprioceptive reflexes and hyperreflexive ataxic hind limb movement responses remained reduced. Over time, there was an improvement in the gains of these reflexes (Table 2). Dog 3 presented syringomyelia prior to MSC transplantation (Figure 3(a)) in the 1st to 6th lumbar vertebrae and demonstrated poor recuperation, with only a discrete panniculus reflex recovery. Dog 2 had a less compressive lesion and demonstrated a hypersignal at the 3rd lumbar (Figure 3(b)). Following MSC therapy, dog 2 was able to sustain a complete weight bearing position for one minute, 100 days after surgery (Figure 4).

These recovery findings occurred simultaneously with the reestablishment of bowel and urinary bladder functions in dogs 1 and 2. Despite slow movement progression, constant improvement was observed over the next 3 months. Complete and partial self-controlled tail movement was observed in dog 1 and dog 3, respectively. After 6 months of transplantation, no additional clinical gains were observed. Dogs 3 and 4 remained with recurring urinary bladder dysfunction and infection due to the incapacity of the weight bearing position, with no observed gains, according to MFS (Table 2) [32].

### 3.3. MRI Evaluation

The MRI diagnosis provided visualization of the entire injured spine, including the vertebral bodies, intervertebral discs, vertebral canal, and spinal cord (Figure 3). The remaining disorders included intervertebral disc degeneration (without compressive intervertebral disk protrusions/extrusion or relevant clinical signals) in three dogs (two cervical and one lumbar). All dogs presented areas of hyperintensity of the spinal cord, greater than or equal to the length of the L2 vertebral body, in T2-weighted magnetic resonance images. Neither mass lesions nor nerve root tumors were observed. One dog presented hydromyelia/syringomyelia (dog 4) and another was identified to have an extradural synovial cyst (Table  3) (dog 3). Extradural compressive lesions were neither detected before nor after cell therapy. Data generated by MRI revealed no changes at the MSC administration site into the spinal cord or within urinary bladder.

## 4. Discussion

The use of naturally diseased dogs is a very interesting option to develop cell-based regenerative strategies, since most canine diseases are functionally and structurally quite similar to those described in humans [15]. In the present study, we evaluated the potential of autologous MSC transplantation to regenerate canine injured spinal cord. We observed that MSC presented *in vitro *properties are quite similar to those observed in humans [33] and other species of laboratory animals [7, 17, 30, 33, 34]. Cultured MSC showed osteogenic and adipogenic differentiation properties and high proliferation indexes, which emphasize the feasibility of its use for therapeutic directive, including the treatment of neurologic disorders [6–10].

Engrafted cells may be a source of necessary secretory factors that counteract the inhibition caused by glial cells and maximize the intrinsic regenerative potential of endogenous progenitor cells. It has been reported that some factors secreted by mesenchymal stem cells support neural differentiation of embryonic stem cells [35]. Since neural progenitor cells express a wide variety of chemokine receptors and migration-related proteins that could potentially influence progenitor cell migration, neurogenesis, and gliogenesis [9], it is possible that, at least in part, the clinical improvements shown by the dogs in the present study can be justified by the interaction between engrafted MSC and endogenous spinal cord-derived factors.

Another potential approach aiming to minimize the progression of secondary injury can be achieved by modulating the neuroinhibitory environment of the spinal cord. It is hypothesized that the application of a scaffold in the surgical wound may avoid scarring and subsequent cyst formation. The mechanism for this is not yet known, but it has been associated with controlling the exaggerated cellular growth by a programmed structure, such as a “net,” where the cells can grow and proliferate [8]. Inflammation-mediated lesion expansion has been correlated with the secondary injury processes [5], where the lesion size is speculated to be correlated with the degree of functional deficit [36]. During the surgical procedure, after applying MSC, we used collagen gel as a scaffold, acting as a biodegradable composite with the purpose to secure the cells within the injured area. However, our purpose was not to anatomically reconstruct the surgical site, as previously reported [8].

Dogs are prone to autoimmune diseases which can be provoked by the introduction of foreign proteins like FCS or FBS-derived molecules. In some cases, immunological reactions and anti-FBS antibodies have been observed and considered to affect the therapeutic outcome [37]. Thus, although in our study we did not observe any immune-mediated reaction in our dogs, alternative supplements could be used for late comparisons with FBS for MSC isolation and expansion. Previously, researches [38] indicate that thrombin-activated platelet releasate plasma and pooled platelet lysate supplements support the isolation and expansion of MSC compared to FBS.

The dogs in the present study presented a faster clinical outcome/recovery in the first 100 days following cell transplantation, but some recovery signs were still observed up to the 18th month of clinical monitoring in each animal. The continuous progression included improvement of the cutaneous trunci muscle reflex on the back skin; however, some recoveries of reflexes that occurred faster in the initial stages were not observed at the end of the 12-month period. This suggests that the major clinical benefits seen following stem cell therapy were due to factor(s) secreted by MSC in a discontinuous manner that have yet to be identified.

Urogenital disorders secondary to SCI include disruption of supraspinal input, afferent input to spinal cord, and reorganization of intraspinal circuitry in response to injury [24–26]. As a result, in most cases, bladder control is partially achieved only if decompression is performed shortly following a severe spinal cord compression in dogs [39]. Previous efforts to restore bladder function, including nerve transplantation, were not successful despite low control over the reinnervation and thus coordinating voiding [40]. In the present work, the clinical improvements in bladder control observed in two dogs were not associated with gain in spinal cord MRI images, which also were sustained throughout the entire recovery period. Two years after transplantation, the clinical bladder gain showed limited improvements and was not associated with any observable long- or short-term complications.

The dogs included in the present study had chronic paraplegia secondary to intervertebral disk herniation and were not expected to regain a normal gait with any other known treatment. Unfortunately, the lack of muscle control was not enough to insure that the canine patients would walk again. One of the largest differences between dogs and human with respect to self-standing remains in locomotion expectations. A smooth diminishing on hypointense areas was observed within SCI by MRI, showing no counter indication of the technique; however, following cell therapy, no imaging benefits were observed by this exam. Using cell therapy in these types of clinical cases has some limitations, as histological data from the spinal cord cannot be generated and therefore direct conclusions regarding tissue benefits and improvements cannot be made.

## 5. Conclusion

In conclusion, bone marrow-derived MSC collecting and administration protocols were safe and relatively simple for therapy of spinal cord injury in dogs. Cell therapy with autologous bone marrow within the injured spinal cord in dogs produced some clear clinical benefits to the animals. Although feasible results were reached, more research is still required for clinical practice and additional research is necessary to standardize the application.

## Figures and Tables

**Table 1:** Characteristics of dogs submitted to MSC therapy.

Dog	Age (years)	Sex	Lesion length	Lesion level
1	2	Female	9 mm	T13-L1
2	4	Male	12 mm	L1-L3
3	3	Male	10 mm	L1-L3
4	2	Male	30 mm	L1-L7

**Table:** (a)

Clinical findings	Dog 1			Dog 2			Dog 3			Dog 4		
	Before MSC	100 d	12 mo	Before MSC	100 d	12 mo	Before MSC	100 d	12 mo	Before MSC	100 d	12 mo
Tail movement	(−)	(−)	Partial gain	(−)	Total gain	Total gain	(−)	(−)	Partial gain	(−)	(−)	(−)
Panicculi reflex	(−)	Partial gain	Partial gain	(−)	(−)	Partial gain	(−)	Partial gain	Partial gain	(−)	Partial gain	Partial gain
Urinary bladder continence	(−)	Partial gain	Partial gain	(−)	Partial gain	Partial gain	(−)	Partial gain	(−)	(−)	(−)	(−)
Propioc. Reflex	(−)	Partial gain	Partial gain	(−)	Partial gain	Partial gain	(−)	(−)	(−)	(−)	(−)	(−)
Weight bearing	(−)	(−)	(−)	(−)	1 min	1 min	(−)	(−)	(−)	(−)	(−)	(−)

**Table:** (b)

	Before MSC	12 mo	Before MSC	12 mo	Before MSC	12 mo	Before MSC	12 mo
MFS	0	1	0	1	0	0	0	0

	Before MSC	18 mo	Before MSC	18 mo	Before MSC	18 mo	Before MSC	18 mo

Rx	0	0	0	0	0	0	0	0

Before MSC: before administration of MSC; 100 d: 100 days after MSC transplantation; 12 mo: 12 months after MSC transplantation; MFS: modified Frankel score; propioc proprioception; Rx: radiogram evaluation (0: neither proliferative/erosive observations nor fracture/luxation of the spine; 1: presence of signs of proliferative/erosive alterations or fracture/luxation of the spine).

**Table 3:** Summary results from magnetic resonance imaging.

	DOG 1		DOG 2		DOG 3		DOG 4	
MRI imaging	Before MSC	12 months after MSC	Before MSC	12 months after MSC	Before MSC	12 months after MSC	Before MSC	12 months after MSC
	Increased	Unchanged	Increased	Unchanged	Increased	Increased and ESC	Increased	H/S

Increased: increased signal intensity; ESC: extradural synovial cyst; H/S: hydromyelia/syringomyelia.
